# Update on the management of relapsed/refractory chronic lymphocytic leukemia

**DOI:** 10.1038/s41408-024-01001-1

**Published:** 2024-02-21

**Authors:** Rory Bennett, John F. Seymour

**Affiliations:** 1 Department of Clinical Haematology, Royal Melbourne Hospital and Peter MacCallum Cancer Centre, 305 Grattan St, Parkville, Melbourne, VIC 3000 Australia; 2 University of Melbourne, Grattan St, Parkville, Melbourne, VIC 3010 Australia

**Keywords:** Chronic lymphocytic leukaemia, Chronic lymphocytic leukaemia, Targeted therapies, Genetic testing

## Abstract

Chronic lymphocytic leukemia (CLL) predominantly affects older adults, characterized by a relapsing and remitting pattern with sequential treatments available for many patients. Identification of progressive/relapsed CLL should prompt close monitoring and early discussion about the next therapies when treatment indications are present. The intervening period represents an opportunity to optimize patient health, including establishing adequate vaccination and surveillance for second primary malignancies, and treating non-CLL-related comorbidities which may impact well-being and CLL therapy. We now see patients with relapsed/refractory (RR) CLL in the clinic who have been previously treated with chemoimmunotherapy (CIT) and/or one or more novel therapies. Continuous covalent inhibitors of Bruton’s tyrosine kinase (cBTKi) and fixed-duration venetoclax (Ven)-anti-CD20 monoclonal antibody (mAb) are preferred over CIT given the survival advantages associated with these therapies, although have never been evaluated head-to-head. While both classes are effective for RR CLL, potential side effects and the logistics of administration differ. Few randomized data demonstrate the sequential use of cBTKi and fixed-duration Ven-anti-CD20 mAb; however, they may be used in either sequence. Newer non-covalent BTKi, active against *BTK* C481 resistance mutations emerging with continuous cBTKi exposure, and novel approaches such as BTK degraders, bispecific antibodies, and chimeric antigen receptor T-cell therapies demonstrate impressive efficacy. In this review of RR CLL we explore relevant investigations, consideration of broader CLL- and non-CLL-related health needs, and evidence for efficacy and safety of B-cell receptor inhibitors and Ven, including available data to support drug sequencing or switching. We describe novel approaches to RR CLL, including rechallenging with fixed-duration therapies, allogeneic stem cell transplant indications in the novel therapy era, and highlight early data supporting the use of T-cell directing therapies and novel drug targets.

## Introduction

Survival outcomes for patients with chronic lymphocytic leukemia (CLL) continue to improve decade on decade, heavily owing to advances in treatment. Availability of novel inhibitors of B-cell receptor signaling (BCR, Bruton’s tyrosine kinase [BTKi] [1] or phosphatidylinositol 3-kinase [PI3ki]) [2] or B-cell lymphoma-2 (BCL2i) [3] has transformed the treatment landscape for treatment-naïve (TN) and relapsed/refractory (RR) CLL. In most developed nations, patients with CLL will likely have access to multiple effective novel therapy options at either first treatment indication and/or upon sequential relapses. The therapeutic armamentarium will likely continue to expand with the emergence of T-cell directing therapies, chimeric antigen receptor T-cell (CAR-T) therapy [4–8], bispecific antibodies (bsAb) [9], and BTK degrader molecules [10, 11] which demonstrate promise in early phase studies treating RR CLL.

With any disease relapse, the disease burden and kinetics and presence of high-risk genomic lesions, and the possibility of Richter transformation should be carefully considered. The window of opportunity between relapse and next-line therapy should be used to optimize CLL-related sequelae and non-CLL-related health issues. When treatment is required, the selection and sequencing of novel therapies for RR CLL should consider individual patient priorities, including tolerance for potential side effects as well as the perceived efficacy of therapy. In this review, we outline the optimal approach to the patient with RR CLL, including a discussion of the evidence for current and emerging treatment options in this setting.

## Assessment of the patient with relapsed/refractory CLL

### Timing of next treatment

Recognition of RR CLL does not necessitate immediate change or initiation of the next therapy. Broadly, criteria-based indications to treat RR CLL should align with iwCLL 2018 treatment indications [12] per TN CLL, however, maintenance of patient well-being and function is the ultimate goal and this should not be compromised by deferral of therapy awaiting formal iwCLL criteria to be met. Upon relapse, it is important to have pre-emptive discussions with patients regarding the perceived timing of further therapy, establish future treatment goals, and consider the frequency of interim clinical and hematologic monitoring.

The impact of prior treatment and biological variables on the kinetics of disease progression can be appreciated in the kinetics of changes in measurable residual disease (MRD). For those attaining undetectable MRD (uMRD) following fixed-duration venetoclax-rituximab (VenR) in the MURANO study, the time from conversion to MRD positivity to fulfillment of iwCLL criteria for the progressive disease was 28.3 months [3, 13]. Shorter median MRD doubling time following VenR was observed for CLL with complex karyotype (CK, ≥3 copy number variants [CNVs]) vs. no CK (*p* = 0.054), and unmutated IGHV (umIGHV) vs. mutated IGHV (mIGHV, *p* = 0.0057) [14]. A pattern of exponential increase in MRD was observed following VenR In contrast to the logistic-type growth pattern following bendamustine-rituximab (BR) [14]. These data suggest potential utility in closer monitoring for those patients with known high-risk genomic lesions where more complex planning for subsequent treatment options may be required.

As for treatment-naïve CLL, auto-immune cytopenia in isolation do not mandate treatment of the underlying CLL, unless a trial of conventional immune suppressants is not successful. Ultimately, the use of ibrutinib (Ib) or Ven appear to successfully improve or resolve secondary auto-immune cytopenia for the majority of treated patients with CLL [15].

Patients with RR CLL needing further treatment who are at increased risk of death may be identified through the validated four-point BALL score [16]. One point is scored for each of (1) serum β_2_-microglobulin ≥5 mg/dL, (2) lactate dehydrogenase above the upper limit of normal, (3) hemoglobin <110 g/L (women)/<120 g/L (men), and (4) time from initiation of last therapy of <24 months, with higher combined scores predictive of greater likelihood of death within 24 months. This model has been validated in cohorts of patients receiving with either Ib, Ven, or Idela (or CIT) [16]. This score is not designed to aid selection between novel therapies but may be used practically when discussing goals of care or when considering the role of allogeneic stem cell transplantation or clinical trials.

### Evaluation of chronic CLL-related complications and other health issues

The work-up of a patient with RR CLL is an opportunity to review disease- and non-disease-related health issues and relevant preventative health measures. CLL is associated with intrinsic B- and T-lymphocyte dysfunction from which multiple important complications of the disease arise, including hypogammaglobulinemia, recurrent infections, and increased incidence of second primary malignancies [17–19]. In addition, many patients may experience significant immunological deficits from previous therapies, which may compound these risks [19].

It is important to ensure adherence to age- and gender-appropriate malignancy screening such as mammography, cervical smear, prostate-specific antigen testing, and colorectal cancer screening. Vigilance to skin protection measures and annual dermatological assessments are essential given the disproportionately augmented rates of non-melanomatous skin cancers (and melanoma) which occur for patients with CLL [17, 20]. It is important to encourage non-live vaccinations against seasonal influenza, *Streptococcus pneumoniae*, and *Varicella zoster* where accessible. Given the current context of the ongoing SARS-CoV-19 pandemic, receipt of vaccinations per local health administration authority recommendations is important. Despite intrinsic immunological dysfunction, adequate seroconversion is achievable with multiple vaccinations [21], and T-cell responses are relatively preserved [22]. Peri-vaccination withholding of BTKi may improve rates of seroconversion but not median anti-spike antibody titers overall [23].

The majority of patients with RR CLL are elderly, with many afflicted by multiple medical issues. Seemingly unrelated but impactful concerns may be discovered in the work-up of RR CLL, including failing cardiovascular health, diabetes and hypertension (HTN), diminished bone density, and, more broadly, frailty or threatened loss of independence. These issues may have a greater bearing on well-being and/or survival than co-existent CLL in older adults [24]. Optimization of broader health in advance of treatment may allow for maintenance of function during therapy and, where relevant, minimize the incidence of complications from novel therapies such as atrial fibrillation/flutter (AF) with BTKi or tumor lysis affected by impaired renal function with Ven.

### Consideration of Richter transformation

Richter transformation (RT) to aggressive B-cell lymphoma forms part of the natural history of CLL, with variable reported incidence between studies. The presence of RT may be clinically or biochemically suspected due to proliferative features more consistent with aggressive B-cell lymphoma, such as new onset fevers, hypercalcemia, discordant focal adenopathy, or involvement of extra-nodal sites. Although ‘false-positives’ are common, dominant lesions with discordant SUVmax >5–10 on FDG-PET should be biopsied to exclude RT [25]. Where possible, a clonal relationship to co-existent CLL should be established through IGHV sequencing or assessment of histological PD-1 expression, a useful surrogate marker of clonal relatedness [26, 27].

### Genomic reassessment: evidence of clonal evolution and acquisition of high-risk genomic abnormalities

High-risk genomic abnormalities have an important bearing on the prognosis of CLL, none more so than aberrations of *TP53* on chromosome 17 (del([17p] and/or *TP53* mutations), which retain prognostic significance in the novel therapy era [3, 28–30].

While the IGHV mutational status does not vary throughout treatment and testing need not be repeated, it is important to re-assess the patient with RR CLL by karyotyping and fluorescence in situ hybridization (FISH), and sequencing of *TP53* if no aberrations were found previously. Prevalence of *TP53* aberrations increases with serial therapies and are overrepresented in patients with fludarabine- and/or double-refractory disease [31]. Clonal evolution by molecular studies may be predicted by the presence of baseline sub-clonal driver mutations and likely portends a poorer prognosis [32]. Significant early clonal shift can be observed with selection pressure from Ib, associated with a greater likelihood of disease progression [33]. However, while the identification of dynamic mutations in recognized driver genes such as *ATM*, *NOTCH1*, and *BIRC3* may be of academic interest but currently does not guide current treatment selection.

Testing the patient with RR CLL for resistance mutations to novel therapies remains largely investigational. Continuous exposure to BTKi or BCL2i may induce resistance mutations of genes coding for respective target proteins [34–36], however, the full clinical utility of identifying variants at disease progression is yet to be established. For example, the presence or absence of *BTK* C481 variants following disease progression on cBTKi would not alter the recommendation not to treat with further cBTKi. Whether next-generation BCL2i may be effective in the presence of *BCL2* variants is currently being evaluated (NCT04277637). In contrast, the incidence of resistance mutations following time-limited novel therapies appears very low [37, 38].

## Current therapeutic strategies for relapsed/refractory CLL

**Fig. 1:**
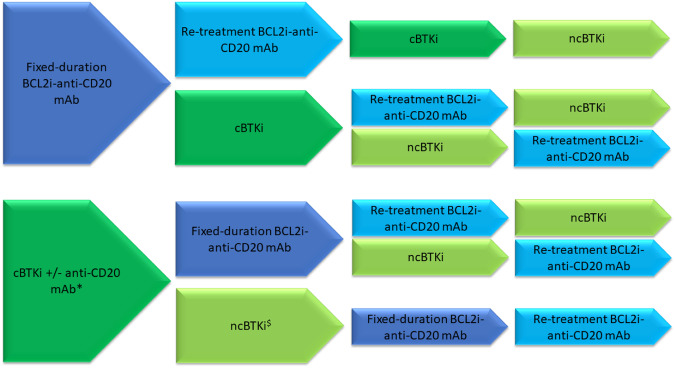
Possible sequencing of novel therapies for patients with RR CLL independent of prior chemoimmunotherapy. *In event of intolerance, a second cBTKi may be considered initially if appropriate. ^$^Current approvals for Pirto are restricted to BTKi- and BCL2i-exposed patients. BCL2iB-cell lymphoma-2 inhibitor, cBTKi covalent Bruton’s tyrosine kinase inhibitor, mAb monoclonal antibody, ncBTKi non-covalent BTKi.

We now see patients requiring second or further therapies following prior CIT or novel therapies or both. We do not recommend repeat use of CIT for RR CLL, given the superior efficacy of novel therapies. With prior CIT only, either cBTKi or BCL2i may be selected; with disease progression after one prior novel therapy exposure, the alternative novel agent class is generally recommended as the next therapy. Exceptions may include an alternative cBTKi in the event of intolerance to initial cBTKi, or consideration of retreatment with fixed-duration combination therapies such as Ven-anti-CD20 mAb after previous fixed-duration therapy with the same. There are no published head-to-head data to inform the efficacy and safety of BTKi vs. BCL2i; hence selection of therapies rests on expert opinion and individualized patient-clinician discussions. A network meta-analysis of novel therapies for untreated CLL did not demonstrate differences in PFS between Ven-obinutuzumab (VenO), IbO, and acalabrutinib (Acala) [39]. Possible novel therapy sequences are depicted in Fig. 1.

Beyond drug access and prior therapies received, several important factors should influence the selection of the next therapy for the patient with RR CLL. It is important to discuss (1) patient goals and priorities, including preferences where present for treatment-free remissions, (2) the perception of treatment destination, whether sequential non-curative strategies are favored, and/or if potential curative allogeneic stem cell transplantation has a role, (3) potential complications in view of comorbidities and concurrent medications, (4) the availability of clinical trials, perception of their merits and effect on the treatment journey.

In this section, we discuss the evidence for novel therapies for RR CLL and the evidence for sequencing classes of novel therapies. Patients with CLL refractory to both cBTKi and BCL2i have unmet therapeutic need—we also discuss emerging novel therapies, including immunotherapies under development.

### Evidence for efficacy and safety of covalent BTK inhibitors (cBTKi)

Final analysis from the RESONATE study at a median of 6.5 years provides the longest follow-up of randomized data describing novel therapies in RR CLL [1]. Once-daily Ib demonstrated markedly superior PFS compared with ofatumumab (HR 0.148 [95% CI 0.113–0.196], *p* < 0.001), with a suggestion of overall survival benefit favoring Ib when censored at study cross-over, *see* Table 1. In recently presented phase II data in long follow-up, PFS rates following Ib were 22.4% after a median of 113 months and OS of 41.6% after a median of 117 months [40]. Twice-daily second-generation cBTKi Acala and Zanu were developed to augment BTK drug occupancy and reduce off-target tyrosine kinase inhibition-associated side effects [34]. Acala demonstrated superior PFS for patients with RR CLL treated with bendamustine-rituximab (BR) in the ASCEND study; [41] whilst Zanu has not been compared with CIT in the RR setting, Zanu-treated patients with untreated CLL without del[17p] demonstrated improved PFS compared with BR, HR 0.42 (95% CI 0.28–0.63, *p* < 0.0001) [42].

**Table 1:** Pivotal phase III studies informing comparative efficacy and safety of novel therapies for RR CLL.

Study/treatment	Study population	Median follow-up	PFS	OS	Cytopenias	Infections	Other/cardiac
GS-1101-0116 Ph III; IdelaR vs. PlboR, *n* = 220 Furman, *NEJM* 2014 Composite primary & secondary studies of Idela-treated patients (*n* = 110)^&^ Shadman, *JCO* 2019	Considered unfit for CIT by investigators Median age; 71 y vs. 71 y Median prior lines; 3 vs. 3 UmIGHV; 83 vs. 85% Del[17p] or *TP53* mut; 42 vs. 45%	^&^18 months	24 weeks; 93 vs. 46% (HR 0.15 [95% CI 0.08–0.28]; *p* < 0.001) ^&^IdelaR -> Idela: mPFS 20.3 months	24 weeks; 92 vs. 80% (HR 0.28 [95% CI 0.09–0.86], *p* = 0.02) ^&^IdelaR -> Idela; mOS 40.6 months ^&^Plbo-R -> Idela; mOS 34.6 months	Grade ≥3:^&^ neutropenia 12.7%, thrombocytopenia NR	IdelaR -> Idela Grade ≥3:^&^ all infections 53.6%, PJP 3.6% (none had received prophylaxis) 22 occurrences of fungal infections	IdelaR -> Idela, grade ≥3:^&^ diarrhea 16.4%, colitis 8.2%, pneumonitis 6.4%, AST rise 5.5%, ALT rise 9.1%, bleeding 6.4% Any-grade AF/flutter 3.6%^&^, no HTN AEs reported AEs resulting in death 11.8%^&^
DUO Ph III; Duvel vs. Ofa, *n* = 319 Flinn, *Blood* 2018	Excluded BTKi/Pi3Ki-treated patients Median age; 69 y vs. 69 y Median prior lines; 2 vs. 2 UmIGHV; 69 vs. 73% Del[17p] and/or *TP53* mut; 31 vs. 33%	22.4 months	mPFS 13.3 months vs. 9.9 months (HR 0.52, *p* < 0.0001)	mOS NR vs. NR 12 m OS 86% for both treatments	Grade ≥3: neutropenia 30 vs. 17%, thrombocytopenia 8 vs. 2%	Any-grade: all infections 69 vs. 43%, 3 pt. vs. 1 pt. PJP infections	Grade ≥3: colitis 12 vs. 1%, ALT rise 3%, AST rise 3% TEAE discontinuation; 35% (Duvel arm) AEs resulting in death 11.9% (Duvel arm)
RESONATE Ph III; Ib vs Ofa, *n* = 391 Byrd, *NEJM* 2014* Munir, *AJH* 2019^#^	Median age; 67 y vs. 67 y Median prior lines; 3 vs. 2 UmIGHV; 73 vs. 63% Del[17p]; 32 vs. 32% *TP53* mut; 51 vs. 46%	65.3 months^#^	mPFS 44.1 months vs 8.1 months (HR 0.148 [95% CI 0.11–0.20], *p* < 0.001) ^#^	mOS 67.7 months vs. 65.1 months^#^ N.B. 68% cross-over to Ib	Grade ≥3: neutropenia 25% (Ib^#^) (16% Ib vs. 14% Ofa*), thrombocytopenia 10%^#^ (6% Ib vs. 4% Ofa*)	Grade ≥3: all infections 24 vs. 22%*, longer f/up – 45% (Ib^#^)	Ib-treated patients: major hemorrhage 10%, any grade AF/flutter 12%, any grade HTN 21% TEAE discontinuation; 16% (Ib)
ELEVATE RR Ph III; Acala vs. Ib, *n* = 533 Byrd, *JCO* 2021	Median age; 66 y vs. 65 y Median prior lines; 2 vs. 2 UmIGHV; 82.1 vs. 89.4% Del[17p]; 45.1 vs. 45.3% *TP53* mut; 37.3 vs. 42.3%	40.9 months	mPFS 38.4 months vs. 38.4 months (HR 1.00 [95% CI 0.79–1.27])	mOS NR vs. NR	Grade ≥3: neutropenia 19.5 vs. 22.8%, thrombocytopenia 9.8 vs. 6.8%	Grade ≥3: all infections 30.8 vs. 30.0%	Major bleeding 4.5 vs. 5.3%, any grade AF/flutter 9.0 vs. 15.6%, any grade HTN 9.4 vs. 23.2% TEAE discontinuation; 14.7 vs. 21.3%
ASCEND Ph III; Acala vs. investigator choice (IdelaR or BR), *n* = 310 Ghia, *Hemasphere* 2022	Median age; 68 y vs. 67 y Median prior lines; 1 vs 2 UmIGHV; 70 vs. 77% Del[17p]; 17 vs. 14% *TP53* mut; 25 vs. 22%	46.5 months (Acala), 45.3 months (IdelaR/BR)	Overall; mPFS NR vs. 16.8 months (HR 0.28 [95% CI 0.20–0.38], *p* < 0.001) Acala vs. IdelaR; mPFS NR vs. 16.2 months (HR 0.30 [95% CI 0.22–0.42], *p* < 0.001) Acala vs. BR; mPFS NR vs 18.6 months (HR 0.24 [95% CI, 0.16 − 0.38], *p* < 0.001)	mOS NR vs. NR (HR 0.69 [95% CI 0.46 − 1.04], *p* = 0.078)	Acala/IdelaR/BR, grade ≥3: neutropenia 19/40/31%, thrombocytopenia 4/8/3%	Acala/IdelaR/BR, grade ≥3: all infections 29/34/11%	Acala/IdelaR/BR: major hemorrhage 3/3/3%, any grade AF/flutter 8/3/3%, any grade HTN 8/6/0% TEAE discontinuation; 23% (Acala), 67% (IdelaR), 17% (BR)
ALPINE Ph III; Zanu vs. Ib, *n* = 652 Brown, *NEJM* 2023	Median age; 67 y vs. 68 y Median prior lines; 1 vs 1 UmIGHV; 73.1 vs. 73.5% Del[17p] and/or *TP53* mut; 22.9 vs 23.1%	29.6 months	mPFS NR vs. 34.2 months (HR 0.65 [95% CI 0.49–0.86], *p* = 0.002)	mOS NR vs. NR (HR 0.76 [95% CI 0.51–1.11])	Grade ≥3: neutropenia 16 vs. 13.9%, thrombocytopenia 2.8 vs. 3.7%	Grade ≥3: all infections 26.5 vs. 28.1%	Major bleeding 3.4 vs. 3.7%, any grade AF/flutter 5.2 vs. 13.3%, any grade HTN 23.5 vs. 22.8% TEAE discontinuation; 15.4 vs 22.2% Cardiac deaths 0 vs. 6
MURANO Ph III; VenR vs. BR, *n* = 389 Seymour, *NEJM* 2018 Seymour, *Blood* 2022^	Median age; 64.5 y vs. 66 y UmIGHV; 68.3 vs. 68.3% Del[17p]; 26.6 vs. 27.2% *TP53* mut; 25.0 vs. 27.7%	59.2 months^	mPFS 53.6 months vs 17.0 months (HR 0.19 [95% CI 0.15–0.26], *p* < 0.0001)^	mOS NR vs. NR (HR 0.4 [95% CI 0.26–0.52], *p* < 0.0001)^	Grade ≥3: neutropenia 57.7 vs. 38.8%, thrombocytopenia 5.7 vs. 10.1%	Grade ≥3: all infections 17.5 vs. 21.8%	Grade ≥3: TLS 3.1 vs. 1.1% Clinical TLS; 1 pt. vs 1 pt. TEAE deaths; 5.2 vs. 5.9%

^&^Secondary study after early termination of primary study due to superior efficacy of IdelaR; patients in either arm could then receive Idela monotherapy.

*AF/flutter* atrial fibrillation/flutter, *CI* confidence interval, *CIT* chemoimmunotherapy, *Duvel* duvelisib, *HR* hazard ratio, *HTN* hypertension, *Ib* ibrutinib, *Idela* idelalisib, *Ofa* ofatumumab, *mOS* median OS, *mPFS* median PFS, *NR* not reached, *Plbo* placebo, *pt* patient, *R* rituximab, *TEAE* treatment-emergent adverse event.

Comparative efficacy of Ib vs Acala and Acala vs Zanu in RR CLL have been evaluated in the large ELEVATE RR [43] and ALPINE studies, respectively, see Table 1. Patients treated with Acala had non-inferior PFS to Ib, including for key subgroups including del[17p], umIGHV and *TP53-*mutated CLL [43]. In contrast, Zanu is associated with PFS benefit compared with Ib (HR 0.67 [95% CI 0.52–0.86]) for all patients in recently present data from ALPINE [44] at three years study follow-up; [45] concern about reduced median duration of response for Ib-treated patients when indirectly compared with previous studies of Ib in RR CLL has been raised [46] and contested [47]. The PFS benefit seen with Zanu compared with Ib was retained for the same key subgroups including *TP53* aberrant CLL, HR 0.52 (95% CI 0.32–0.83) [45].

All cBTKi are effective therapies for RR CLL agnostic of genomic risk, although high-risk abnormalities may influence efficacy. Early studies of Ib have identified the presence of del[17p] [48] and *NOTCH1* and *TP53* mutations [49] as independent predictors of inferior PFS. In exploratory analyses of Ib-treated patients from RESONATE, patients with *TP53* aberrations (*n* = 104) observed median PFS of 40.6 months (95% CI: 27.5–44.1), whereas median PFS for patients without del[11q] nor *TP53* aberrations (*n* = 58) was not reached [1]. Similarly, while the PFS benefit of Acala was sustained compared to IdelaR/BR-treated patients, the median PFS for patients with *TP53* aberrant CLL was 45.5 months, and not reached for patients without del[17p] and/or *TP53* mutation [43].

BTKi are associated with several class-based side effects which are overall reduced with second-generation cBTKi. Compared with Ib, Acala was associated with less any-grade AF and HTN, less any-grade bleeding but with similar rates of major hemorrhage in ELEVATE RR [43], and lower burden of AEs overall in a treatment-exposure adjusted post hoc analysis [50]. No difference in any-grade HTN or any-grade bleeding/major hemorrhage, but less any-grade AF occurred on Zanu compared with Ib in ALPINE [44]. Rates of adverse-event-related treatment discontinuation were higher with Ib than either Acala, or Zanu (21.3–22.2% vs. 14.7%, 15.4% respectively at primary analyses) [43, 44]. A major concern with Ib is the incidence of ventricular arrhythmia and sudden cardiac death in key studies of TN and RR CLL [1, 37, 51–54]. Presented data from a pooled study of five prospective trials has not demonstrated increased ventricular arrhythmias or sudden cardiac death with Acala [55]. Most events occur early within Ib therapy, and most affected patients have preexisting cardiovascular comorbidities.

Overall, cBTKi are effective therapies for RR CLL with well-described and manageable toxicity profiles. From an efficacy standpoint, all available agents are likely similar, although Zanu may provide more durable PFS in comparison to Ib, including for patients with *TP53* aberrant CLL. With respect to minimizing toxicities, Acala or Zanu may be preferable to Ib.

### Evidence for non-covalent BTKi (ncBTKi)

Non-covalent BTKi reversibly bind BTK and remain active in *BTK* C481 mutated clones observed at CLL progression after cBTKi. Pirto was evaluated for cBTKi-exposed patients with RR CLL (*n* = 282) in the phase 1/2 BRUIN study [56] leading to recent FDA approval for cBTKi and BCL2i-treated patients. Recently updated follow-up data presented include ORR (including PR-L) 82% with a median PFS 19.4 months after a median 27.5 months follow-up. Although numerically inferior response rates and duration of response were observed for BCL2i-exposed patients (compared with BCL2i-naïve), these patients were more heavily pretreated (median five vs. three prior lines) [57]. Pirto appears well tolerated with low rates of grade ≥3 AF and HTN and TEAE-related treatment discontinuation (2.5%) [56, 57]. In a recent matching-adjusted indirect comparison of Pirto vs VenR for cBTKi-treated patients, no difference in PFS or OS was observed between the two approaches (although higher ORR and fewer grade ≥3 AEs were observed with Pirto) [58]. Nemtabrutinib (Nemta) has a somewhat less favorable safety profile but with similar efficacy data in phase II studies for RR CLL (BELLWAVE-001) [59]. Newer ncBTKi AS-1763 [60] and novel covalent/non-covalent BTKi LP-168 [61] are under evaluation in ongoing studies.

Fixed-duration Pirto-Ven(R) has demonstrated high ORR and promising 18-month PFS rates of >80% for patients with RR CLL in presented data from a phase 1b arm of the BRUIN study [62]. Phase III studies combining Pirto-VenR vs. VenR (NCT04965493) for patients with CLL previously treated with cBTKi and Nemta-VenR vs. VenR (NCT05947851) for patients with CLL previously treated with one line of therapy are underway.

Overall, Pirto is an effective agent approved for use after cBTKi and BCL2i exposure and appears well tolerated with low incidence of TEAE.

### Evidence for venetoclax (BCL2i)

Ven is the first-in-class BCL2i with efficacy as continuous monotherapy and as fixed-duration combination therapy with Ritux over a total of 24 months [3, 63]. While ORR are similar between the approaches, rates of complete response (CR) and uMRD were indirectly higher in a phase 1b study of venetoclax-rituximab (VenR) as compared with the first-in-human study of Ven monotherapy (CRR 51%, 57% uMRD vs. pooled CRR 20%, uMRD CR in 5% patients) [63, 64]. The phase III MURANO study demonstrated superior PFS and OS for VenR compared with BR for patients with RR CLL, the majority of whom had one prior line of therapy, *see* Table 1 [3]. In updated data, the 7-year PFS rate was 23.0% following VenR with a median time-to-next-treatment of 63 months [13].

We now have a comprehensive understanding of factors predicting durable responses or early failure following Ven. Achieving uMRD with time-limited VenR is associated with longer PFS and OS [3, 65], and depth of MRD response is predictive of the durability of response; 3-year PFS estimates from completion of therapy were 61.3 vs 40.7% for uMRD vs low-MRD positivity (10^−4^ to <10^−2^) [14]. VenR demonstrates meaningful PFS for all high-risk subgroups, however, PFS following VenR was shorter for RR CLL with umIGHV, *TP53* aberrancy, and genomic complexity (≥3 copy number alterations) [14]. A phase II study of Ven monotherapy for patients with del[17p] CLL only, ORR was 77% with an mDoR of 39.3 months (RR CLL) [66, 67]. In a pooled analysis of 347 patients with RR CLL receiving 400 mg Ven monotherapy, additional factors associated with shorter durability of response were refractoriness to prior BCRi and increased lymph node size (≥5 cm and especially ≥10 cm) [65].

VenR is a well-tolerated treatment and minimizes cumulative adverse events due to delivery as fixed-duration therapy. It is initiated in dose ramp-up due to risks of tumor lysis syndrome (TLS) which can be managed effectively by risk stratification and prophylactic TLS measures including the use of rasburicase, intravenous fluids, and close observation [3, 64]. Key safety outcomes from MURANO are summarized in Table 1.

Overall, fixed-duration VenR induces deep responses and the potential toxicities are both well understood and readily mitigated. Shorter durations of response may be expected for patients with genomically high-risk disease.

### Evidence for efficacy and safety of phosphatidylinositol 3-kinase inhibitors (PI3Ki)

Idelalisib (Idela) and duvelisib (Duvel), disrupt B-cell receptor signaling through inhibition of phosphatidylinositol 3-kinase (PI3K) δ and δγ, respectively [68]. While Idela(-R) [2] and Duvel [69] have demonstrated PFS benefits compared with anti-CD20 mAb. IdelaR demonstrated inferior PFS to Acala in the ASCEND study [41], *see* Table 1. Median PFS remains less than 2 years for any PI3Ki-treated patients in available randomized data [2, 69].

High rates of TEAE-related treatment discontinuation, including fatal AE (8.4% [DUO] and 11.8% ([GS-1101-0116]), although the minority of fatalities were attributed to drugs [2, 41, 69]. The toxicity profiles are characterized by frequent infections (including *Pneumocystis jirovecii* pneumonia and cytomegalovirus reactivation), and immune-mediated colitis, pneumonitis, and hepatitis, amongst others.

Overall, PI3ki appear directly and indirectly inferior to BTKi with respect to efficacy, toxicity, and treatment discontinuation [70]. These agents are, therefore, less preferable to BTKi (or BCL2i) where available.

### Sequencing of therapies for RR CLL

Both cBTKi are BCL2i-anti-CD20 mAb are highly effective therapies for RR CLL. With independent mechanisms of action and resistance, these therapies may be used sequentially in either order [71, 72]. There are limitations to this understanding, including a lack of randomized data informing the efficacy of both possible sequences, a lack of comprehensive distinction between novel therapy-exposed and -refractory patients, and that most patients with RR CLL in pivotal studies have previously received CIT.

Prospective data evaluating Ven -> cBTKi include 14 patients in MURANO who received BTKi following progression after fixed-duration VenR, all of whom responded to BTKi therapy. In a retrospective cohort of BTKi-naïve patients receiving cBTKi after Ven discontinuation (*n* = 42; 73% received Ven monotherapy), the median PFS was 32 months from BTKi initiation [73]. Other smaller retrospective series have reported similar efficacy [71]. For cBTKi -> Ven; an open-label phase II study of 91 patients received Ven monotherapy after Ib discontinuation (due to PD [55%], intolerance [33%], or other) reported ORR/CRR 65%/9% and median PFS of 24.7 months [72]. The ORR to Ven monotherapy following ibrutinib failure was 79% in a real-world study of novel agent sequencing [74]. In recent retrospective series of cBTKi-treated patients enriched for high-risk genomic lesions, PFS and TTNT following VenR appear shorter than those reported in MURANO (in which five patients were cBTKi-exposed) [75, 76]. Single-agent Ib appears effective following progression after fixed-duration Ib-Ven [77].

Selecting initial novel therapy requires individualized patient-physician discussion, considering treatment goals and priorities, including duration of therapy and logistical concerns, and the potential side effects in the context of known comorbidities. Fixed-duration VenR may be favored for potential treatment-free remissions and minimization of accrued treatment-related toxicities [78]. Logistical drawbacks include weekly dose escalation with frequent venipuncture and potential for intermittent inpatient observation, and intravenous administration of anti-CD20 mAb. VenR is not suitable for patients with severe renal dysfunction. In contrast, BTKi are continuous oral therapies which do not require dose titration or similarly intensive initial monitoring. Continuous therapy may be problematic due to the perceived “pill burden” to the patient, cumulative toxicities leading to treatment discontinuation, and selection of resistant clones through continuous selection pressure. BTKi may be challenging to deliver for patients with preexisting cardiovascular comorbidities as discussed in a previous section.

Retreatment with BCL2i-anti-CD20 mAb may be considered after previous fixed-duration therapy as dynamic resistance mechanisms, including *BCL2* mutations are unlikely to have developed [14, 37, 38, 77, 79]. In a MURANO sub-study, 25 patients received VenR treatment at a median of 2.3 years from the last Ven dose with an ORR of 72%, and a median PFS of 23.3 months from retreatment [13]. Undetectable MRD responses were comparatively transient compared with initial treatment. A retrospective series of Ven-re-treated patients (*n* = 46, 40% cBTKi-exposed, predominantly RR CLL) demonstrated similar ORR (79%) and mPFS (25 months) with re-treated with 41.7% uMRD responses [80]. These findings question whether future analyses of time-to-next-treatment following fixed-duration Ven therapies should incorporate attempts at retreatment where appropriate [29]. An actively recruiting study seeks to explore the merits of this approach following first-line VenO (NCT04895436).

For those patients who are intolerant of one cBTKi, it is feasible to commence an alternative cBTKi as the next therapy [81–83]. Most pre-defined intolerance events either do not recur or recur with less severity for patients treated with Acala following Ib intolerance [81, 83], or Zanu following either Acala or Acala [84] and Ib intolerance [82]. The estimated 24-month PFS for patients with RR CLL treated with Acala following Ib was 72% [83]. Intolerance due to ventricular arrhythmias or major hemorrhage may be compelling reasons not to consider a second cBTKi.

There are unanswered questions about the potential future sequencing of BTK-targeting therapies. The discovery of “dead-kinase” *BTK* variants (e.g., L528W) following Zanu with the demonstration of subsequent reduced Pirto efficacy has generated concern that ncBTKi may not be effective following Zanu [85]. Additionally, the acquisition of non-C481 “dead-kinase” *BTK* resistance mutations following Pirto may generate cross-resistance to second-generation cBTKi [86], which could impact consideration of ncBTKi as the inaugural BTK-targeting therapy. However, the true incidence of ‘dead-kinase’ mutations following selective cBTKi and ncBTKi is largely unknown, and as such, these concerns are presently theoretical.

### Emerging novel therapies for RR CLL

#### Small molecules and antibodies

Degraders of the BTK protein are an emerging class of therapy with potential utility for patients previously treated with both BTKi and BCL2i, including those with *BTK* resistance mutations. Preliminary data from the first human studies of BTK degraders NX-2127 [10], BGB-16673 [11], and NX-5948 [87] demonstrated early efficacy in RR non-Hodgkin lymphoma, including R/R CLL.

ROR1 is not expressed by normal B-cells and has been targeted with a monoclonal antibody (cirmtuzumab); however, best responses have been limited to stable disease in a small number of patients with CLL [84]. Following incomplete response to or resistance mutation to BTKi, anti-BAFF antibodies (Ianalumab, VAY736) are proposed to deepen responses (including uMRD) [88].

The phase 1b/2 study of novel MCL1 AZD5991 monotherapy for RR hematological malignancies (including five patients with CLL) closed prematurely due to a high incidence of laboratory troponin elevation, with concurrently low response rates [89].

### T-cell directing therapies

Although CLL was successfully treated in the first clinical reports with CAR-T, the development of T-cell-directing therapies has been comparatively slow compared with NHL, likely owing to the availability of effective novel therapies.

Recent early-phase data for CAR-T demonstrate promising ORR and rates of bone marrow (BM) uMRD, including limited patients in high-risk genomic subgroups [4, 6, 8]. Efficacy and limited safety data from recent key early studies of autologous CAR-T and bsAb in RR CLL are summarized in Table 2. A minority of patients may experience durable remissions from CAR-T with the persistence of CAR-T cells now reported for up to 10 years, provoking the question of whether these patients have been cured of CLL [90]. Likelihood of durable response appears associated with depth of response, including uMRD [4–6]. However, reported PFS outcomes are relatively short for all patients. Rates of CRS appear high compared with non-Hodgkin lymphoma, likely owing to the higher burden of circulating and/or BM disease present. Fatal TEAE, including CRS and neurotoxicity, have been described [4–8].

**Table 2:** Key early phase studies of immunotherapies in RR CLL.

Study/treatment	Population	Median follow-up	ORR/CR(i)R (%), uMRD	PFS/OS	mDoR/mDoCR	Any/≥Grade 3 CRS (%)	Any/≥Grade 3 neurotoxicity	Other
NCT01747486 Ph II; autologous CTL019 (tisagenlecleucel) low-dose vs. high-dose, enrolled/infused/response evaluable, *n* = 42/38/32 Frey, *JCO* 2020	Median age; 61.6 y Median prior lines; 3.5 *TP53* aberrant; 28% UmIGHV; 72% Prior BTKi exposure; 28% Prior venetoclax exposure; 3%	31.5 months	44%/28%	mPFS/mOS; 1 month/64 months	Not reported	63%/24% (Penn Grading scale), 59%/11% (ASTCT)	x/7.9%	No significant association between response and patient age, number of prior therapies, stage at enrollment, *TP53* aberrancy or IGHV mutation status. Median PFS was 57.1 months (CR group) and 2.8 months (PR group), p = 0.0013. 1 fatal TEAE (infection)
NCT02640209 Ph II; autologous CTL019 (huCAR-T19) added to Ib (best response <CR), enrolled/infused/response evaluable, *n* = 20/19/18 Gill, *Blood Advances* 2022	Median age; 62 y Median prior lines; 2 *TP53* aberrant – 60% Prior BTKi exposure – 100%	42 months	x/44%, 72.2% uMRD at 12 months	48 month estimates 70/84%	Not reported	94.7/10.6% (ASTCT)	26.3/5.3%	Median 14 months on Ib prior to CAR-T CAR-T; 5/19 and 14/19 received 2 and 3 infusions 2 fatal TEAE - 1 CRS + ICANS, 1 ?due to Ib.
NCT03331198 (TRANSCEND 004) Ph I/II; open-label autologous liso-cel; Apheresed/infused, *n* = 137/117 Siddiqi, *Lancet* 2023*	Median age; 65 y Median prior lines; 5 88% BTKi refractory, 76% Ven refractory, 60% double-refractory Bulky (>5 cm) lesions – 44% High-risk genomics (CK, TP53 aberrancy, and/or IGHV unmutated) – 84%.	21.1 months	43%/18%, uMRD 63% (blood)/59% (BM)	mPFS/mOS; 11.9 months/30.3 months	35.3 months/not reached	85%/9%	45%/19%	Primary endpoint of CRR (>null hypothesis) was met, but not secondary endpoint ORR. ORR/CR similar for TP53 aberrant and double-refractory patients. DoR PR vs. CR, 23.8 months vs. not reached. In post hoc analyses, uMRD associated with superior PFS irrespective of best overall response achieved. 5 fatal TEAE – one considered related to liso-cel
NCT03331198 (TRANSCEND 004) Ph I/II; liso-cel and Ib combination cohort, infused *n* = 19 Wierda, *Blood* 2020	Median age; 60 y Median prior lines; 4 100% BTKi refractory, 74% as last therapy Prior venetoclax; 53% exposed	Not reported	95%/47%, uMRD 89% (flow)/79% (NGS) in BM	Not reported	Not reported	74/5.3%	32/15.8%	15/18 responders maintained response at 3/12 follow-up No fatal TEAE
NCT01865617 Ph I/II (CLL and/or Richter transformation); autologous JCAR014; Infused/response evaluable, *n* = 49/47 Liang, *Blood Advances* 2023	Median age; 61 y Median prior lines; 5 100% BTKi refractory Prior venetoclax exposure; 39% High-risk genomics (CK, and/or del[17p]) – 94% Richter transformation – 18% (14% prior, 4% current)	79.6 months	70%/16%, uMRD 70% (flow) in BM	mPFS/mOS; 8.9 months/25 months	18.9 months/not reported	82/14%	33/27%)	Concurrent ibrutinib associated with higher median peak CD4 + CAR-T expansion. Longer PFS associated with- D + 28 CR and uMRD, higher CD8+ and CD4 + CAR-T-cell expansion, longer CAR-T persistence. Pre-LD bulky disease associated with inferior PFS. Longest measured CAR-T persistence of 86.0 months. Four patients (9%) died in CR/PR, none attributed to CAR-T.
NCT01865617 Ph I/II; (CLL + /-RT) Concurrent Ib and CAR-T (JCAR014) cohort, *n* = 19 infused. Gauthier, *Blood* 2020.	Median age; 65 y High-risk genomics (CK and/or del[17p]); 89% All Ib-refractory; 12 still receiving Ib at study enrollment.	12 months	83%/22%, uMRD by flow 72%/61%	1 year estimates 38/64%	Not reported	74/0%	26/26%	Early death with arrythmia during CRS (Ib related AE). Increased depth of response and uMRD associated with Improved PFS and OS. 32% required Ib dose reduction or discontinuation. Less severe CRS c.f no Ib cohort but no difference in neurotox severity, ?due to reduced tumor burden prior to LD without reduction in peak CAR-T expansion.
NCT04623541 (EPCORE CLL-1) Ph Ib/II; epcoritamab monotherapy - results of dose-expansion, *n* = 23 Kater, *iwCLL* 2023	Median age; 72 Median prior lines; 4 All BTKi exposed, 83% double-exposed *TP53* aberrancy; 65% UmIGHV; 70%	9.3 months	82%/33%	9 month estimates 67/81%	Not reported	95/17%	ICANS 13/0%	3 fatal TEAE

*Primary analysis of monotherapy at Ph II dose.

*BCL2i* B-cell lymphoma-2 inhibitor, Bruton’s tyrosine kinase inhibitor,*CK* complex karyotype, *CRR* complete response (rate), *Do(C)R* duration of (complete) response, *Ib* ibrutinib, *PR* partial response, *uMRD* undetectable measurable residual disease, *TEAE* treatment-emergent adverse event.

Less data are available for bsAb; however, preliminary efficacy is promising. In addition to presented data for CD20xCD3 bsAb from EPCORE CLL-1 [9] (*see* Table 2), objective responses were observed for small numbers of patients with RR CLL (and MCL) treated with time-limited NVG-111 (ROR1xCD3 bsAb) [91].

The limitations of T-cell directing therapies in CLL are incompletely understood; however, much focus is directed toward improving autologous T-cell fitness. Intrinsic CLL-related T-cell dysregulation manifests with impaired T-cell proliferation and immunological synapse formation and may be compounded by prior cytotoxic therapies [19]. The immune-suppressive nodal microenvironment may contribute to the relative persistence of nodal disease following CAR-T compared with clearance of BM disease [92]. Several groups have explored the potential merits of concurrent ibrutinib and CAR-T (in largely cBTKi-refractory patients) in an attempt to restore T-cell fitness and for favorable effect on the nodal microenvironment, *see* Table 2 [7, 93]. Adjunctive approaches to epcoritamab are also planned in the EPCORE CLL-1 study (NCT04623541). Allogeneic CAR-T (NCT04030195, NCT03774654, NCT05878184, NCT 05643742) and CAR-NK products (NCT05739227, NCT05487651, NCT05020678) are also under current evaluation.

### Allogeneic stem cell transplantation

Allogeneic stem cell transplantation (alloSCT) is a potentially curative therapy for high-risk CLL, although usage is restricted to younger fitter patients due to non-relapse mortality (NRM), primarily through infection or graft versus host disease. In long follow-up, studies within the CIT era demonstrate durable 5–10 year remissions for 30–40% of heavily pretreated patients enriched for high-risk genomic abnormalities, but with NRM reported as high as 36% despite the use of reduced-intensity conditioning [94, 95].

Optimal patient selection for alloSCT amongst high-risk CLL is complex. The perceived risk of NRM may be guided by age and comorbidities and can be formally calculated by the HCT-CI score. It is essential to discuss the potential merits and risks in both the short- and long-term to understand whether alloSCT fits within the patient’s treatment priorities and future goals.

While defined initially by the European Bone Marrow Transplantation Committee in 2007 [96], the definition of high-risk CLL is challenging in the novel agent era. With widely available novel therapies and improved survival outcomes for patients with *TP53* aberrant CLL, “high-risk” may be refined dynamically by younger age with a short response to the first novel agent, and by diminished availability of sequential therapies. Abysmal overall survival outcomes for patients with “double-refractory” CLL and improved post-alloSCT outcomes with deeper remissions at the time of alloSCT [97] provide some guidance for the potential timing of alloSCT. For high-risk patients, we suggest that alloSCT should be considered in the second deep response (e.g., to the second novel therapy). Optimal therapeutic strategies at progression following upfront combinations therapies like BTKi-BCL2i doublets or BTKi-BCL2i-anti-CD20 mAb triplets are yet to be defined, but alloSCT should also be considered in the second response to retreatment with one or both therapies. While alternative immunotherapies like CAR-T and bsAb have considerable promise, as discussed in the previous section, all are presently investigational, and their curative potential is unknown.

## Conclusions

We are now fortunate to have multiple effective therapeutic options for patients with RR CLL with tolerable safety profiles. With emerging classes of therapy arise new challenges in optimal use and effective sequencing of treatments. Complete care for the patient with RR CLL includes optimization of health factors ostensibly unrelated to CLL, consideration of infection prophylaxis measures and early identification of secondary malignancies, and early discussions about life goals and priorities with assessment of factors which may drive the kinetics of progressive disease.
